# Dependence- and Disability-Free Life Expectancy Across Eight Low- and Middle-Income Countries: A 10/66 Study

**DOI:** 10.1177/0898264319825767

**Published:** 2019-01-30

**Authors:** A. Matthew Prina, Yu-Tzu Wu, Carolina Kralj, Daisy Acosta, Isaac Acosta, Mariella Guerra, Yueqin Huang, Amuthavalli T. Jotheeswaran, Ivonne Z. Jimenez-Velazquez, Zhaorui Liu, Juan J. Llibre Rodriguez, Aquiles Salas, Ana Luisa Sosa, Martin Prince

**Affiliations:** 1 King’s College London, UK; 2 Universidad Nacional Pedro Henriquez Ureña, Santo Domingo, Dominican Republic; 3 National Institute of Neurology and Neurosurgery of Mexico, Mexico City, Mexico; 4 National Autonomous University of Mexico, Mexico City, Mexico; 5 Instituto de la Memoria y Desordenes Relacionados, Lima, Perú; 6 Peking University, Beijing, China; 7 World Health Organization, Geneva, Switzerland; 8 University of Puerto Rico, San Juan, Puerto Rico; 9 Medical University of Havana, Cuba; 10 Universidad Central de Venezuela, Caracas, Venezuela

**Keywords:** life expectancy, disability, dependence, low- and middle-income countries

## Abstract

**Objective:** The objective of this study was to estimate healthy life expectancies in eight low- and middle-income countries (LMICs), using two indicators: disability-free life expectancy (DFLE) and dependence-free life expectancy (DepFLE). **Method:** Using the Sullivan method, healthy life expectancy was calculated based on the prevalence of dependence and disability from the 10/66 cohort study, which included 16,990 people aged 65 or above in China, Cuba, Dominican Republic, India, Mexico, Peru, Puerto Rico, and Venezuela, and country-specific life tables from the World Population Prospects 2017. **Results:** DFLE and DepFLE declined with older age across all sites and were higher in women than men. Mexico reported the highest DFLE at age 65 for men (15.4, *SE* = 0.5) and women (16.5, *SE* = 0.4), whereas India had the lowest with (11.5, *SE* = 0.3) in men and women (11.7, *SE* = 0.4). **Discussion:** Healthy life expectancy based on disability and dependency can be a critical indicator for aging research and policy planning in LMICs.

During this century, the number of people aged 60 and above will have increased dramatically going from 600 million in year 2000 to over 3 billion by 2100. Although this increase will affect every continent, low- and middle-income countries (LMICs) will see a 238% increase in people aged 60 and above, compared with a relatively lower increase of 94% in high-income countries (United Nations, 2015). The rapid growth of aging populations will lead to a rise in chronic illnesses, which are already the main contributors to disease burden in both high- and low- and middle-income countries (Global Burden of Disease Study 2013 Collaborators, 2015). Another major issue associated with both aging and the rise in chronic illnesses is dependence, which has been described as “the need for frequent human help or care beyond that habitually required by a healthy adult” (Harwood, Sayer, & Hirschfeld, 2004). Although some progress has been made in the generation of empirical data regarding the prevalence and risk factors of dependence (At et al., 2015; Sousa et al., 2010), more work is needed, as there is still limited information on its prevalence, particularly in LMICs.

The increase in life expectancy across the world should be celebrated as an achievement, but adding years to life becomes problematic if these years are “unhealthy,” creating further pressure on public health systems and societies. Healthy life expectancy (HLE) indicators have been created as a way to combine disease and mortality information, in order to monitor more effectively whether the years of life gained are spent in a good state of health or not. The majority of research so far has focused on disability-free life expectancy (DFLE), because of the wider availability of appropriate survey data recording this information. Although many studies have reported DFLE in individual LMICs (Brønnum-Hansen, Duraidi, Qalalwa, & Jeune, 2015; Eguez-Guevara & Andrade, 2015; Islam et al., 2017; Lau, Johnson, & Kamalanabhan, 2012; Luo et al., 2016; Moreno et al., 2018) or cross-country comparison (Payne, 2018), few have comparable data across countries and estimated the prevalence of disability using a consistent study design and measurement methods. Previous studies using the World Health Organization Study on Global Aging and Adult Health (SAGE) and the Survey on Health, Well-Being, and Aging in Latin America and the Caribbean (SABE) have suggested variations in DFLE across older adults from various LMICs and highlighted gender differences, where women spent more time in disability than men (Chirinda & Chen, 2017; Minicuci et al., 2011). Given the variety of social and economic development in LMICs, it is important to estimate DFLE from different countries, using comparable data, which could provide more comprehensive evidence.

An indicator that has not received quite as much attention is dependence-free life expectancy (DepFLE), which is somewhat related to “active life expectancy” (Laditka & Ladika, 2009) or severe disability-free life expectancy (Jagger et al., 2016), and is based on care needs. Both dependency and disability are closely related, as disability is often the main cause of dependence, yet not everyone who is disabled has needs for care. Previous research using the 10/66 study data investigated factors contributing to dependence and disability in older age (Sousa et al., 2009; Sousa et al., 2010). Dementia, stroke, limb impairment, and depression were identified to be key contributors to dependence and disability, but the prevalence of these chronic conditions and their population attributable fraction estimates varied across countries (Sousa et al., 2009; Sousa et al., 2010).

DepFLE and DFLE are also likely to be connected, given the close relationship between disability and dependence, but arguably DepFLE could be a more intuitive indicator for policy makers. Few studies have tried to capture both indicators in a range of different low- and middle-income settings, by measuring both disability and dependence outcomes using the same standardized methodology.

The aim of this study is to calculate and report HLE in eight countries in Latin America and Asia in both men and women, by using two indicators: DFLE and DepFLE.

## Method

To calculate healthy life expectancy, we used estimates of the prevalence of disability and dependence from the prevalence phase of the 10/66 Dementia Research Group (DRG) surveys. The 10/66 is a large cohort study, examining health, social, and biological characteristics of older adults living in 12 different sites across eight countries (China, Cuba, Dominical Republic, India, Mexico, Peru, Puerto Rico, and Venezuela; Prina et al., 2017; Prince et al., 2007). Each site, which covered a specific catchment area, contributed between 1,000 and 3,000 participants to the study. The selection of catchment areas was based on accessibility and the networks between the local research groups and community stakeholders. All households were allocated identification numbers, and the interviewers visited all households in the catchment area to identify eligible participants aged 65 or above on a census date. Response rates were excellent across all centers, varying from 72% in urban India to 98% in rural India. The baseline phase was conducted between 2004 and 2006 in all sites apart from Puerto Rico (2007-2010). Each assessment was standardized and validated in each center, allowing for easy comparison of prevalence estimates.

The 10/66 cohort included 17,031 participants across all the sites. This study excluded 41 participants without complete information on age and gender (*N* = 16,990).

### Disability and Dependence Measurements

Two separate approaches were taken to estimate the prevalence of disability in each site. Disability was assessed using the World Health Organization’s Disability Assessment Scale (WHODAS II; Chisolm, Abrams, McArdle, Wilson, & Doyle, 2005). This scale, which assesses 12 different activities (Supplementary Information S1), was specifically developed as a cross-cultural tool to assess levels of disability according to definitions of the International Classification of Functioning, Disability and Health (ICF; World Health Organization [WHO], 2001). Each item is coded with a scale ranging from 0 (*no difficulty*) to 4 (*extreme difficulty*). Standardized scores range from 0 to 100 and were dichotomized using the 90th percentile of the WHODAS II, which indicates the 10% most disabled among the study population (Sousa et al., 2009; Von Korff et al., 2008). In addition to the 12 activities, WHODAS II also includes two single questions on how many days a person was totally unable (1 day) or partially unable (0.5 day) to carry out their usual activities or work because of any health condition in a month. Based on the sum of these two questions, severe disability was defined as having 15 days or more of disability in the previous month of the assessment. This method has been used by our group in several publications (Guerra et al., 2009; Prina et al., 2011).

Dependence was assessed directly during the interview with a family member or key person/caregiver of the participant and was based on the interviewer’s perception of needs for care. The informants were administered a series of open-end questions and more detailed information is provided in Supplementary Information S2. The coding of severity of dependence was based on the “interval of need” (Sousa et al., 2010), which is the length of time during which the person can subsist without human assistance. This interval was then defined by the 10/66 Dementia Research Group as those requiring no care (fully independent) and those needing care much of the time (at least daily) or some of the time (less often than daily). Participants were coded as dependent if they needed care some or much of the time. The percentage of missing data was low in both disability (5.6%) and dependence measures (2.2%).

### Calculations of DFLE and DepFLE

We calculated DFLE and DepFLE for each site using the Sullivan’s method (Sullivan, 1971). This divides life expectancy into healthy (e.g., disability and/or dependence free) and unhealthy years (e.g., disabled and/or dependent), by applying the age- and sex-specific prevalence of the relevant health states to period life tables (EURO-REVES, 2001). Life tables for the eight countries were obtained from the World Population Prospects 2017 (United Nations, 2017). To estimate the life tables which corresponded to year of the 10/66 investigation, data on two periods, 2000-2005 and 2005-2010, were extracted and used to interpolate specific estimates for 2005.

DFLE and DepFLE were estimated by country, age group, and gender in a tabular and a graphic format. Approximate standard errors were calculated taking only the variance of the prevalence rates into account and ignoring the variance of the mortality rates. This approach was suggested by Newman and colleagues (Newman, 1988). Finally, the proportion of remaining life spent free of disability/dependence was calculated by dividing the DFLE/DepFLE by the total life expectancy. We calculated DFLE based on days of disability per month as this definition seems to be more comparable across countries. We also conducted a sensitivity analysis using the 90th percentile of WHODAS II scores and estimated DFLE. The results are reported in Supplementary Information S3.

### Ethics, Consent, and Permissions

The study protocol and the consent procedures, including the witnessed consent procedure, were approved by the King’s College London research ethics committee and in all local countries: (a) Medical Ethics Committee of Peking University the Sixth Hospital (Institute of Mental Health, China); (b) the Memory, Depression Institute and Risk Diseases (IMEDER) Ethics Committee (Peru); (c) Finlay Albarran Medical Faculty of Havana Medical University Ethical Committee (Cuba); (d) Hospital Universitario de Caracas Ethics Committee (Venezuela); (e) Ethics Committee of Nnamdi Azikiwe University Teaching Hospital (Nigeria); (f) Consejo Nacional de Bioética y Salud (CONABIOS, Dominican Republic); (g) Christian Medical College (Vellore) Research Ethics Committee (India); (h) Instituto Nacional de Neurología y Neurocirugía Ethics Committee (Mexico); (i) Nnamdi Azikiwe University Teaching Hospital Nnewi Anambra State Ethics Committee (Nigeria).

## Results

Among the 16,990 participants, 62% were women and the proportion of five age groups were 30% in 65-69, 27% in 70-74, 20% in 75-79, 13% in 80-84% and 10% in 85+. The overall prevalence of disability and dependence was 15% and 10%, respectively. Table 1 reports the prevalence of people with disability and dependence across the different countries, stratified by age group and sex. The prevalence of disability and dependence increased with older age and the trends were consistent across countries. Women generally had higher prevalence of both disability and dependence, in particular in the oldest age group.

**Table 1.** Prevalence of Disability and Dependence Across Sites, Stratified by Age Group and Sex.

	65-69		70-74		75-79		80-84		85+	
	M	F	M	F	M	F	M	F	M	F
Disability										
China (*n* = 2,144)	4.3	4.6	5.5	7.5	11.9	8.9	13.4	17.6	15.8	30.6
Cuba (*n* = 2,896)	6.3	12.2	10.3	17.9	9.0	18.6	20.6	24.4	32.2	42.5
Dominican Republic (*n* = 2,005)	9.0	10.5	13.3	11.7	11.4	14.7	14.1	21.1	21.1	36.1
India (*n* = 1,811)	11.0	15.6	11.5	15.1	9.6	14.7	10.5	26.6	26.5	23.3
Mexico (*n* = 1,980)	8.1	7.1	5.9	5.9	9.8	15.2	14.9	15.9	17.2	20.4
Peru (*n* = 1,488)	13.8	19.7	23.1	26.8	25.0	23.8	26.9	41.7	44.9	55.8
Puerto Rico (*n* = 2,000)	9.3	11.6	12.3	12.7	14.1	18.1	17.5	20.1	32.4	43.3
Venezuela (*n* = 1,719)	5.1	9.0	7.4	8.1	4.8	11.7	13.0	25.8	37.0	30.5
Dependence										
China (*n* = 2,162)	4.6	3.3	5.8	8.5	17.0	12.2	20.4	23.3	28.1	44.6
Cuba (*n* = 2,589)	2.9	3.0	2.2	8.1	5.4	6.6	16.8	16.0	25.9	38.9
Dominican Republic (*n* = 2,005)	4.3	2.6	7.2	8.4	12.1	7.9	15.2	17.0	15.8	42.7
India (*n* = 1,967)	2.6	3.8	4.7	5.7	5.2	5.6	10.0	17.9	13.2	18.4
Mexico (*n* = 2,002)	3.7	5.0	7.7	6.9	8.4	11.2	11.6	14.9	20.0	31.5
Peru (*n* = 1,930)	4.2	1.9	4.7	4.3	7.7	7.4	8.1	19.0	19.6	29.4
Puerto Rico (*n* = 1,996)	6.4	4.3	4.5	6.0	12.2	10.0	15.9	21.2	34.3	46.1
Venezuela (*n* = 1,959)	3.3	3.9	5.7	6.8	12.7	15.1	17.0	24.3	25.8	48.1

*Note.* Disability refers to individuals experiencing 15 or more days of disability in the past month and dependence to individuals needing some or much care most of the time. M = men; F = women.

### DFLE

**Figure 1.**
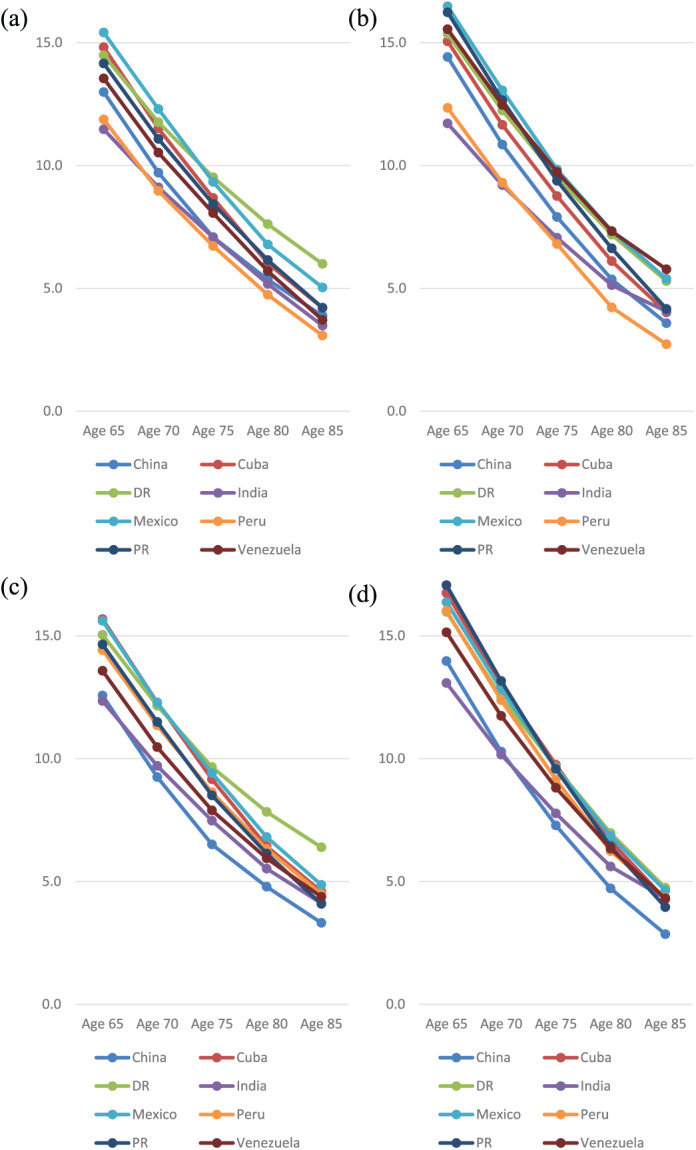
Disability-free life expectancy in men (a) and women (b) by country and age group, and dependence-free life expectancy in men (c) and women (d) by country and age group. *Note.* Disability was assessed using the “more than 15 disability days in the past month” criteria. Dependence was assessed by needing some or much care. DR = Dominical Republic; PR = Puerto Rico.

Table 2 and Figure 1a, 1b report estimated DFLE and the proportion of remaining life spent in disability free. DFLE gradually declined with increasing age but in older age men overtook women in the number of years estimated to be spent free of disability. Among the eight countries, Mexico and Dominican Republic reported higher DFLE while lower estimates were found in India and Peru across all age. Mexico reported the highest DFLE at age 65 for both men (15.4, *SE* = 0.5) and women (16.5, *SE* = 0.4). The lowest was found in India with 11.5 years (*SE* = 0.3) in men and 11.7 (*SE* = 0.4) in women.

**Table 2.** DFLE and Proportion of Remaining Life Sent Disability Free (%) by Country, Age Group, and Gender.

	Age 65		Age 70		Age 75		Age 80		Age 85	
	M	F	M	F	M	F	M	F	M	F
China										
DFLE (*SE*)	13.0 (0.3)	14.4 (0.3)	9.7 (0.2)	10.8 (0.3)	7.1 (0.2)	7.9 (0.3)	5.3 (0.2)	5.4 (0.3)	4.6 (0.2)	3.6 (0.3)
%	92.0	89.3	90.1	86.8	86.9	83.4	85.7	76.8	84.3	69.5
Cuba										
DFLE (*SE*)	14.8 (0.4)	15.0 (0.5)	11.5 (0.4)	11.6 (0.4)	8.7 (0.3)	8.8 (0.3)	6.0 (0.3)	6.1 (0.3)	4.2 (0.3)	4.0 (0.2)
%	86.6	78.1	83.8	74.9	80.9	71.7	73.8	66.0	68.1	57.8
Dominican Republic										
DFLE (*SE*)	14.5 (0.6)	15.3 (0.5)	11.8 (0.5)	12.2 (0.4)	9.5 (0.4)	9.5 (0.4)	7.6 (0.4)	7.2 (0.4)	6.0 (0.4)	5.3 (0.3)
%	86.9	82.0	85.3	79.4	84.6	75.6	82.1	70.2	78.9	63.9
India										
DFLE (*SE*)	11.5 (0.3)	11.7 (0.4)	9.1 (0.3)	9.2 (0.4)	7.1 (0.3)	7.1 (0.4)	5.2 (0.3)	5.1 (0.4)	3.5 (0.3)	4.1 (0.3)
%	88.1	82.7	87.6	81.8	86.9	79.8	83.3	74.9	73.6	76.8
Mexico										
DFLE (*SE*)	15.4 (0.5)	16.5 (0.4)	12.3 (0.4)	13.1 (0.4)	9.3 (0.4)	9.8 (0.4)	6.8 (0.3)	7.3 (0.3)	5.0 (0.3)	5.4 (0.3)
%	89.9	88.1	89.1	86.4	86.6	83.0	84.0	81.8	83.1	79.8
Peru										
DFLE (*SE*)	11.9 (0.7)	12.3 (0.7)	9.0 (0.6)	9.3 (0.6)	6.7 (0.5)	6.8 (0.5)	4.7 (0.5)	4.2 (0.4)	3.1 (0.3)	2.7 (0.3)
%	76.4	69.3	72.2	65.3	69.5	61.5	65.0	51.4	55.2	44.3
Puerto Rico										
DFLE (*SE*)	14.1 (0.6)	16.2 (0.6)	11.1 (0.5)	12.7 (0.5)	8.4 (0.4)	9.4 (0.4)	6.1 (0.3)	6.6 (0.3)	4.2 (0.3)	4.2 (0.3)
%	84.8	79.5	82.4	76.7	79.7	72.3	75.2	67.0	67.7	56.7
Venezuela										
DFLE (*SE*)	13.5 (0.4)	15.5 (0.5)	10.5 (0.4)	12.5 (0.5)	8.1 (0.4)	9.7 (0.5)	5.7 (0.5)	7.3 (0.6)	3.7 (0.5)	5.8 (0.4)
%	89.8	84.2	87.4	81.8	84.4	77.5	75.6	71.5	62.9	69.5

*Note.* DFLE was calculated using “15 or more days of disability in the past month” as its indicator of disability. DFLE = disability-free life expectancy; M = men; F = women.

**Figure 2.**
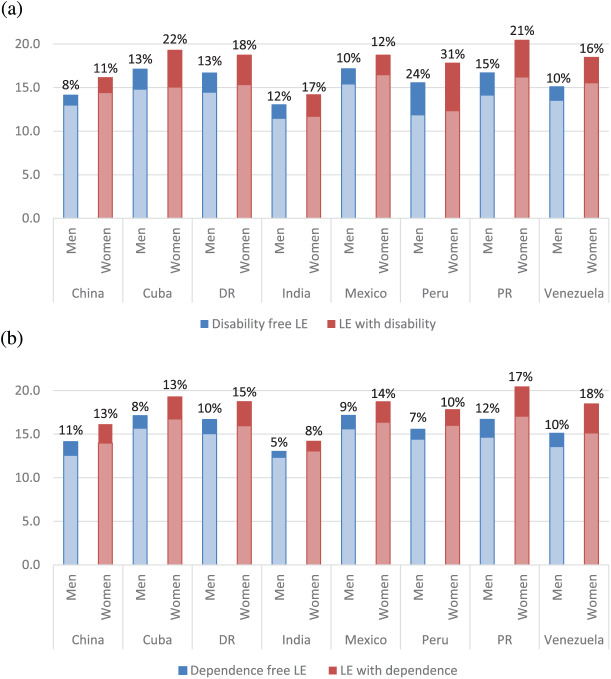
Proportion of life expectancy at age 65 spent with (a) disability or (b) dependence by men and women. *Note.* Disability was assessed using the “more than 15 disability days in the past month” criteria. Dependence was assessed by needing some or much care.

The proportion of remaining life spent disability free (Table 2 and Figure 2a) highlighted that even though women have longer life expectancies, they also tend to spend a longer period of time with disability. This pattern was seen across all countries and the difference increased in older age, apart from the India and Venezuela. Peru had the lowest proportion of remaining life spent disability free for both men (76%) and women (69%) at age 65 and China had the highest (92% and 89% for men and women respectively). In India, where we found the lowest DFLE in absolute terms, a relatively high proportion of remaining life spent disability free was found in men (88%) and women (83%).

Similar results were found when DFLE was calculated using the 90th percentile of WHODAS II scores but variation across countries was smaller (Supplementary Information S3). India still had the lowest DFLE than other sites.

### DepFLE

Similar to DFLE, DepFLE also declined with increasing age across countries (Table 3 and Figure 1c, 1d). Compared with men, women had longer DepFLE in younger age but this was reversed in older old. The longest DepFLE was found in Puerto Rico for women (17.1, *SE* = 0.4) and Cuba for men (15.7, *SE* = 0.3) with Mexico and Peru following closely. India had the lowest DepFLE for both men (12.3, *SE* = 0.2) and women (13.1, *SE* = 0.2).

**Table 3.** DepFLE and Proportion of Remaining Life Sent Dependence Free (%) by Country, Age Group, and Gender.

	Age 65		Age 70		Age 75		Age 80		Age 85	
	M	F	M	F	M	F	M	F	M	F
China										
DepFLE (*SE*)	12.6 (0.3)	14.0 (0.3)	9.2 (0.3)	10.3 (0.3)	6.5 (0.3)	7.3 (0.3)	4.8 (0.3)	4.7 (0.3)	3.3 (0.3)	2.9 (0.3)
%	89.0	86.6	85.8	82.3	79.9	76.9	76.6	67.5	72.0	55.5
Cuba										
DepFLE (*SE*)	15.7 (0.3)	16.7 (0.3)	12.3 (0.3)	13.0 (0.3)	9.2 (0.3)	9.7 (0.3)	6.4 (0.3)	6.6 (0.3)	4.6 (0.3)	4.3 (0.2)
%	91.6	86.9	89.5	83.5	85.3	79.9	78.8	71.8	74.4	61.5
Dominican Republic										
DepFLE (*SE*)	15.0 (0.5)	16.0 (0.4)	12.1 (0.4)	12.5 (0.4)	9.7 (0.4)	9.6 (0.3)	7.8 (0.4)	7.0 (0.3)	6.4 (0.3)	4.7 (0.3)
%	90.3	85.3	88.1	81.2	85.8	76.6	84.5	68.1	84.2	57.3
India										
DepFLE (*SE*)	12.3 (0.2)	13.1 (0.2)	9.7 (0.2)	10.2 (0.3)	7.5 (0.2)	7.8 (0.3)	5.5 (0.3)	5.6 (0.3)	4.1 (0.2)	4.3 (0.3)
%	94.8	92.3	93.3	90.4	91.9	87.9	88.8	81.9	87.0	81.6
Mexico										
DepFLE (*SE*)	15.6 (0.4)	16.4 (0.4)	12.3 (0.4)	12.8 (0.4)	9.4 (0.3)	9.6 (0.4)	6.8 (0.3)	6.8 (0.3)	4.9 (0.3)	4.6 (0.3)
%	91.0	87.5	89.0	84.9	87.3	81.2	84.3	76.4	80.3	68.6
Peru										
DepFLE (*SE*)	15.5 (0.3)	16.0 (0.3)	11.4 (0.3)	12.4 (0.3)	8.6 (0.3)	9.1 (0.3)	6.3 (0.2)	6.2 (0.3)	4.5 (0.2)	4.3 (0.3)
%	92.7	90.0	91.4	87.0	89.2	82.7	86.7	76.0	80.5	70.7
Puerto Rico										
DepFLE (*SE*)	14.6 (0.5)	17.1 (0.4)	11.5 (0.4)	13.2 (0.4)	8.5 (0.4)	9.6 (0.4)	6.1 (0.3)	6.4 (0.3)	4.1 (0.3)	4.0 (0.3)
%	87.8	83.5	85.5	79.7	80.3	73.9	75.0	65.0	65.7	53.9
Venezuela										
DepFLE (*SE*)	13.6 (0.4)	15.1 (0.5)	10.5 (0.4)	11.7 (0.5)	7.9 (0.4)	8.8 (0.5)	5.9 (0.5)	6.3 (0.5)	4.4 (0.5)	4.3 (0.4)
%	90.0	82.0	87.0	77.1	82.6	70.1	78.9	61.9	74.2	51.9

*Note.* DepFLE is based on individuals needing some or much care. DepFLE = dependence-free life expectancy; M = men; F = women.

Despite higher life expectancy, women spent a higher proportion of time with care needs and the gender difference increased with older age (Table 3 and Figure 2b). Women in Venezuela (52%), Puerto Rico (54%), China (56%), and Dominican Republic (57%) had less than 60% dependence-free time while men still had 65% or above. Although India had the lowest DepFLE at age 65, it was estimated to have the highest proportion of life spent in dependence free in both men (95%) and women (92%) and these high estimates remained in the oldest age group. Peru, where the proportion of remaining life spent disability-free was the lowest in both men and women, ranked relatively high when looking at dependence-free at age 65. However, the proportion largely decreased with older age.

## Discussion

This study investigated DFLE and DepFLE in adults aged 65 and above, across eight countries in Latin America and Asia. Although women had longer life expectancies, they also spent more time in a state of disability and dependence. DFLE and DepFLE decreased with increasing age across all countries. Estimates using year 2000 and year 2011 life tables were relatively comparable, and so were the estimates when disability was measured using the 90th percentile of WHODAS II scores.

### Limitations

Although this is one of the largest studies measuring both DFLE and DepFLE across a large number of countries, some limitations have to be acknowledged. Estimates in the latter age groups (85 and above) may not be as stable as the ones in younger age groups, because of increased attrition in our samples and consequent widening of our confidence intervals. Our samples, even though carefully selected to be as representative as possible of the general population, are unlikely to be as comprehensive as those from a nationally representative sample. Some LMICs such as China and India have large numbers of older people but the 10/66 study only included a small sample from specific areas in different country. Although the overall percentage of missing disability and dependence measures were low, some sites had relatively high numbers of missing data than others. Finally, we used life tables as close as possible to the year of the surveys, but this still meant an average 5-year gap between the year of the life tables (2011) and the one from our surveys (2003-2006). We do not expect the prevalence of disability and dependence to have gone up significantly in 5 years, but to explore this in more depth we also decided to present a sensitivity analysis using year 2000 life tables, effectively bracketing our data.

### Contextualization

Most of the HLE indicators have mainly focused on DFLE, with little research done on DepFLE particularly in LMICs. This indicator, which is closely related to DFLE, is particularly relevant for policy purposes, as it directly links to care needs, which is ultimately associated with economic burden. A recent study using the Cognitive Function and Ageing Studies (CFAS), population-based cohorts of older adults in England, United Kingdom, has investigated levels of dependency based on the interval of care needs (Isaacs & Neville, 1976) and suggested increases in years lived with low (less than daily) and high (24-hr care) dependency over the last two decades (Kingston et al., 2017). It was estimated that only 64% men and 47% women at age 65 lived independently in England (Kingston et al., 2017). These estimated proportions of DepFLE seem to be much lower than our study but such differences might be attributed to variation in measurement methods of care needs.

We have previously shown that caregiving is associated with adverse economic impacts upon the household, manifesting primarily through larger health care costs and reduction in paid work for the caregiver to care (Acosta et al., 2010; Z. R. Liu et al., 2009; Uwakwe et al., 2009). An understanding of the economic and social implications of care dependence among some of these countries will become clearer once the results from the INDEP study are published (Mayston et al., 2014).

Using the same sampling design, standardized measurement instruments and wording across all of our sites allow us to increase the comparability and reliability of our findings. Previous studies, such as the Survey of Health and Retirement in Europe (SHARE) (Jagger et al., 2011) have estimated health expectancies across countries but have mainly focused on high-income countries. The current state of evidence on healthy life expectancies in low- and middle-income countries is still relatively small and mainly focuses on DFLE. The results from our Latin American sites are comparable to those reported by Eguez-Guevara and colleagues who carried out a study on life expectancy in Ecuador using limitations in activities of daily living (ADL) as their measure of disability (Eguez-Guevara & Andrade, 2015). DFLE at age 65 was estimated to be 13.6 years in men and 13.1 years in women, which are similar to the results we presented from bordering Peru. DFLE for year 2000 was also measured in the SABE study which was conducted in six sites in Latin America in Bridgetown, Sao Paulo, Santiago, Havana, Mexico City, and Montevideo (Minicuci et al., 2011). DFLE Estimates for Bridgetown (Barbados) of 16.6 years in women and 14.9 in men at age 65 were very close to the ones we reported for Puerto Rico. The estimates for Havana (Cuba) were also relatively close in both studies, whereas the ones in Mexico City (Mexico) were lower in the SABE study compared with ours. This is probably due to the fact that the SABE study only sampled a major urban centre, compared with our two sites in Mexico, and also used 2000 estimates. The SAGE study, including nearly 35,000 older adults from in China, Russia, Ghana, Mexico, South Africa and India, has estimated DFLE at age 50 and three of these countries (China, Mexico and India) overlapped with our study. Despite differences in baseline age and disability measures, both SAGE and our study found that India had the shortest DFLE compared with other LMICs (Chirinda & Chen, 2017).

HLE has also been previously calculated in China and India, even though studies from the Asian continents are scarcer. Using disability data from the 2001 census and life tables from the WHO, Lau and colleagues (2012) reported year 2010 DFLE in India for both men and women that were very similar to the ones we presented. Finally, a recent publication using data from the China Health and Retirement Longitudinal Study (CHARLS) reported life expectancy in China using several different states of health, including good perceived health, chronic diseases, active life, and severe impairment (Luo et al., 2016), updating the previous estimates of J. Liu, Chen, Song, Chi, and Zheng (2009).

Some of the cross-country variations may be explained by the wide prevalence of chronic diseases across the various countries and their contribution to disability and dependence (Sousa et al., 2009; Sousa et al., 2010).

## Conclusion

This study presents new evidence on DFLE and DepFLE across a large number of low- and middle-income countries. This type of evidence is needed, especially in low- and middle-income countries, where the largest percentage of people living with severe disabilities lives (WHO, 2011). Future research should also focus on understanding drivers and trajectories of healthy life expectancies, and our new study, LIFE2YEARS (Prina et al., 2017), will provide a solid starting platform to explore drivers and trends. It is important that health expectancies are monitored over time to understand trends across regions. The WHO (2015) in their world report on aging & health urged that improving measurement and monitoring of healthy aging are key areas of action. We believe that dependency is a critical indicator, ideal for future research planning and for the assessment of large complex interventions.

## Supplemental Material

sullivan_paper_-_suppl – Supplemental material for Dependence- and Disability-Free Life Expectancy Across Eight Low- and Middle-Income Countries: A 10/66 Study Click here for additional data file. Supplemental material, sullivan_paper_-_suppl for Dependence- and Disability-Free Life Expectancy Across Eight Low- and Middle-Income Countries: A 10/66 Study by A. Matthew Prina, Yu-Tzu Wu, Carolina Kralj, Daisy Acosta, Isaac Acosta, Mariella Guerra, Yueqin Huang, Amuthavalli T. Jotheeswaran, Ivonne Z. Jimenez-Velazquez, Zhaorui Liu, Juan J. Llibre Rodriguez, Aquiles Salas, Ana Luisa Sosa and Martin Prince in Journal of Aging and Health

## References

[bibr1-0898264319825767] Acosta D. Rottbeck R. Rodriguez J. G. Gonzalez L. M. Almanzar M. R. Minaya S. N.. . . Prince M. J. (2010). The prevalence and social patterning of chronic diseases among older people in a population undergoing health transition: A 10/66 group cross-sectional population-based survey in the Dominican Republic. BMC Public Health, 10, Article 344. doi:10.1186/1471-2458-10-344 PMC2953638 20553582

[bibr2-0898264319825767] At J. Bryce R. Prina M. Acosta D. Ferri C. P. Guerra M.. . . Prince M. (2015). Frailty and the prediction of dependence and mortality in low- and middle-income countries: A 10/66 population-based cohort study. BMC Medicine, 13, Article 138. doi:10.1186/s12916-015-0378-4 PMC4481121 26063168

[bibr3-0898264319825767] Brønnum-Hansen H. Duraidi M. Qalalwa K. Jeune B. (2015). Increasing disability-free life expectancy among older adults in Palestine from 2006 to 2010. European Journal of Public Health, 25, 335-339. doi:10.1093/eurpub/cku069 24906845

[bibr4-0898264319825767] Chirinda W. Chen H. (2017). Comparative study of disability-free life expectancy across six low- and middle-income countries. Geriatrics & Gerontology International, 17, 637-644. doi:10.1111/ggi.12748 27197085

[bibr5-0898264319825767] Chisolm T. H. Abrams H. B. McArdle R. Wilson R. H. Doyle P. J. (2005). The WHO-DAS II: Psychometric properties in the measurement of functional health status in adults with acquired hearing loss. Trends Amplification, 9, 111-126. 10.1177/108471380500900303 PMC4111522 16244758

[bibr6-0898264319825767] Eguez-Guevara P. Andrade F. C. (2015). Gender differences in life expectancy with and without disability among older adults in Ecuador. Archives of Gerontology and Geriatrics, 61, 472-479. doi:410.1016/j.archger.2015.1008.1012 26316253 10.1016/j.archger.2015.08.012

[bibr7-0898264319825767] EURO-REVES. (2001). Health expectancy calculation by the Sullivan method: A practical guide. European Health Expectancy Monitoring Unit.

[bibr8-0898264319825767] Global Burden of Disease Study 2013 Collaborators. (2015). Global, regional, and national incidence, prevalence, and years lived with disability for 301 acute and chronic diseases and injuries in 188 countries, 1990-2013: A systematic analysis for the Global Burden of Disease Study 2013. The Lancet, 386, 743-800. doi:10.1016/S0140-6736(15)60692-4 PMC4561509 26063472

[bibr9-0898264319825767] Guerra M. Ferri C. P. Sosa A. L. Salas A. Gaona C. Gonzales V. de la Torre G. R. Prince M (2009) Late-life depression in Peru, Mexico and Venezuela: the 10/66 population-based study. British Journal of Psychiatry, 195(6), 510-515. doi: 10.1192/bjp.bp.109.064055 19949200 PMC2915389

[bibr10-0898264319825767] Harwood R. H. Sayer A. A. Hirschfeld M. (2004). Current and future worldwide prevalence of dependency, its relationship to total population, and dependency ratios. Bulletin of the World Health Organization, 82, 251-258. 15259253 PMC2585969

[bibr11-0898264319825767] Isaacs B. Neville Y. (1976). The needs of old people: The “interval” as a method of measurement. British Journal of Preventive and Social Medicine, 30, 79-85. 953380 10.1136/jech.30.2.79 PMC478944

[bibr12-0898264319825767] Islam M. S. Tareque M. I. Mondal M. Fazle Rabbi A. M. Khan H. Begum S. (2017). Urban-rural differences in disability-free life expectancy in Bangladesh using the 2010 HIES data. PLoS ONE, 12(7), e0179987. 28742101 10.1371/journal.pone.0179987 PMC5524410

[bibr13-0898264319825767] Jagger C. Matthews F. E. Wohland P. Fouweather T. Stephan B. C. Robinson L.. . . Brayne C. (2016). A comparison of health expectancies over two decades in England: Results of the Cognitive Function and Ageing Study I and II. The Lancet, 387, 779-786. doi:10.1016/s0140-6736(15)00947-2 PMC4761658 26680218

[bibr14-0898264319825767] Jagger C. Weston C. Cambois E. Van Oyen H. Nusselder W. Doblhammer G.. . . Robine J. M. (2011). Inequalities in health expectancies at older ages in the European Union: Findings from the Survey of Health and Retirement in Europe (SHARE). Journal Epidemiology & Community Health, 65, 1030-1035. doi:10.1136/jech.2010.117705 21471138

[bibr15-0898264319825767] Kingston A. Wohland P. Wittenberg R. Robinson L. Brayne C. Matthews F. E., . . . Cognitive Function and Ageing Studies collaboration. (2017). Is late-life dependency increasing or not? A comparison of the Cognitive Function and Ageing Studies (CFAS). The Lancet, 390, 1676-1684. 10.1016/S0140-6736(17)31575-1 PMC5640505 28821408

[bibr16-0898264319825767] Laditka S. B. Ladika J. N. (2009). Active life expectancy: A central measure of population health. doi:10.1007/978-1-4020-8356-3_24

[bibr17-0898264319825767] Lau R. S. Johnson S. Kamalanabhan T. J. (2012). Healthy life expectancy in the context of population health and ageing in India. Asia-Pacific Journal of Public Health, 24, 195-207. doi:10.1177/1010539510376663 20685664

[bibr18-0898264319825767] Liu J. Chen G. Song X. Chi I. Zheng X. (2009). Trends in disability-free life expectancy among Chinese older adults. Journal of Aging and Health, 21, 266-285. doi:10.1177/0898264308328978 19104033

[bibr19-0898264319825767] Liu Z. R. Albanese E. Li S. R. Huang Y. Q. Ferri C. P. Yan F.. . . Prince M. (2009). Chronic disease prevalence and care among the elderly in urban and rural Beijing, China—A 10/66 dementia research group cross-sectional survey. BMC Public Health, 9, Article 394. doi:10.1186/1471-2458-9-394 PMC2770493 19843346

[bibr20-0898264319825767] Luo H. Wong G. H. Lum T. Y. Luo M. Gong C. H. Kendig H. (2016). Health expectancies in adults aged 50 years or older in China. Journal of Aging and Health, 28, 758-774. doi:10.1177/0898264315611663 26491044

[bibr21-0898264319825767] Mayston R. Guerra M. Huang Y. Sosa A. L. Uwakwe R. Acosta I.. . . Prince M. J. (2014). Exploring the economic and social effects of care dependence in later life: Protocol for the 10/66 research group INDEP study. SpringerPlus, 3, 379. doi:10.1186/2193-1801-3-379 25105086 PMC4124109

[bibr22-0898264319825767] Minicuci N. Noale M. Leon Diaz E. M. Gomez Leon M. Andreotti A. Mutafova M. (2011). Disability-free life expectancy: A cross-national comparison among Bulgarian, Italian, and Latin American older population. Journal of Aging and Health, 23, 629-681. doi:10.1177/0898264310390940 21220352

[bibr23-0898264319825767] Moreno X. Albala C. Lera L. Leyton B. Angel B. Sánchez H. (2018). Gender, nutritional status and disability-free life expectancy among older people in Santiago, Chile. PLoS ONE, 13(3), e0194074. 29590148 10.1371/journal.pone.0194074 PMC5874002

[bibr24-0898264319825767] Newman S. C. (1988). A Markov process interpretation of Sullivan’s index of morbidity and mortality. Statistics in Medicine, 7, 787-794. 3406606 10.1002/sim.4780070708

[bibr25-0898264319825767] Payne C. F. (2018). Aging in the Americas: Disability-free life expectancy among adults aged 65 and older in the United States, Costa Rica, Mexico, and Puerto Rico. The Journals of Gerontology. Series B, Psychological Sciences and Social Sciences, 73, 337-348. doi:10.1093/geronb/gbv076 PMC6283317 26347520

[bibr26-0898264319825767] Prina A. M. Ferri C. P. Guerra M. Brayne C. Prince M (2011). Co-occurence of anxiety and depression amongst older adults in low- and middle-income countries: findings from the 10/66 study. Psychological Medicine, 41(10), 2047-2056. doi: 10.1017/S0033291711000444 21466747

[bibr27-0898264319825767] Prina A. M. Acosta D. Acostas I. Guerra M. Huang Y. Jotheeswaran A. T.. . . Prince M. (2017). Cohort profile: The 10/66 study. International Journal of Epidemiology, 46, 406-406i. doi:10.1093/ije/dyw056 27154633 PMC5837706

[bibr28-0898264319825767] Prince M. Ferri C. P. Acosta D. Albanese E. Arizaga R. Dewey M.. . . Uwakwe R. (2007). The protocols for the 10/66 dementia research group population-based research programme. BMC Public Health, 7, Article 165. doi:10.1186/1471-2458-7-165 PMC1965476 17659078

[bibr29-0898264319825767] Sousa R. M. Ferri C. P. Acosta D. Albanese E. Guerra M. Huang Y. Q.. . . Prince M. (2009). Contribution of chronic diseases to disability in elderly people in countries with low and middle incomes: A 10/66 dementia research group population-based survey. The Lancet, 374, 1821-1830. 10.1016/S0140-6736(09)61829-8 PMC2854331 19944863

[bibr30-0898264319825767] Sousa R. M. Ferri C. P. Acosta D. Guerra M. Huang Y. Jacob K.. . . Prince M. (2010). The contribution of chronic diseases to the prevalence of dependence among older people in Latin America, China and India: A 10/66 dementia research group population-based survey. BMC Geriatrics, 10, Article 53. doi:10.1186/1471-2318-10-53 PMC2923155 20691064

[bibr31-0898264319825767] Sullivan D. F. (1971). A single index of mortality and morbidity. HSMHA Health Reports, 86, 347-354. 5554262 PMC1937122

[bibr32-0898264319825767] United Nations. (2015). World population prospects 2015. Retrieved from http://www.un.org/en/development/desa/population/events/other/10/index.shtml

[bibr33-0898264319825767] United Nations. (2017). World population prospects 2017. Retrieved from https://population.un.org/wpp/Download/Standard/Population/

[bibr34-0898264319825767] Uwakwe R. Ibeh C. C. Modebe A. I. Bo E. Ezeama N. Njelita I.. . . Prince M. J. (2009). The epidemiology of dependence in older people in Nigeria: Prevalence, determinants, informal care, and health service utilization. A 10/66 dementia research group cross-sectional survey. Journal of the American Geriatrics Society, 57, 1620-1627. doi:10.1111/j.1532-5415.2009.02397.x 19682135

[bibr35-0898264319825767] Von Korff M. Crane P. K. Alonso J. Vilagut G. Angermeyer M. C. Bruffaerts R.. . . Ormel J (2008). Modified WHODAS-II provides valid measure of global disability but filter items increased skewness. Journal of Clinical Epidemiology, 61, 1132-1143. doi:10.1016/j.jclinepi.2007.12.009 18619808 PMC3277915

[bibr36-0898264319825767] World Health Organization. (2001). International classification of functioning, disability and health. Geneva, Switzerland: Author.

[bibr37-0898264319825767] World Health Organization. (2011). World report on disability. Geneva, Switzerland: Author.

[bibr38-0898264319825767] World Health Organization. (2015). World report on ageing and health. Geneva, Switzerland: Author.

