# Supplementary material for: Dependence- and Disability-Free Life Expectancy Across Eight Low- and Middle-Income Countries: A 10/66 Study
Source: J Aging Health. 2019 Jan 30;32(5-6):401–9. doi: 10.1177/0898264319825767 (PMC7322974; doi:10.1177/0898264319825767)
Supplement: sullivan_paper_-_suppl – Supplemental material for Dependence- and Disability-Free Life Expectancy Across Eight Low- and Middle-Income Countries: A 10/66 Study [file sullivan_paper_-_suppl.pdf]

Dependence and disability free life expectancy across eight low and middle income countries: a 10/66 study

Supplementary Information

S1. WHODAS 2.0 (12 item version)

In the past 30 days, how much difficulty did you have in:

1. Standing for long periods such as 30 minutes?
2. Taking care of your household responsibilities?
3. Learning a new task, for example, learning how to get to a new place?
4. How much of a problem did you have joining in community activities (for example, festivities, religious or other activities) in the same way as anyone else can?
5. How much have you been emotionally affected by your health problems?
6. Concentrating on doing something for ten minutes?
7. Walking a long distance such as a kilometre [or equivalent]?
8. Washing your whole body?
9. Getting dressed?
10. Dealing with people you do not know?
11. Maintaining a friendship? None Mild Moderate Severe Extreme
12. Your day-to-day work?

- Overall, in the past 30 days, how many days were these difficulties present?
- In the past 30 days, for how many days were you totally unable to carry out your usual activities or work because of any health condition?
- In the past 30 days, not counting the days that you were totally unable, for how many days did you cut back or reduce your usual activities or work because of any health condition?

S2. Open-ended questions to key informants in the 10/66 study

- Who shares the home with the participant?
- What kind of help does the participant need inside and outside of the home?
- Who, in the family, is available to care for the participant?
- What help do you provide?
- Do you help to organise care for the participant?
- Is there anyone else in the family who is more involved in helping than you? What do they do?
- What about friends and neighbours? What do they do?

S3. Disability free life expectancy: the 90th percentile of WHODAS 2.0

		Age 65		*Age 70*		*Age 75*		*Age 80*		*Age 85*	
		M	***F***	***M***	***F***	***M***	***F***	***M***	***F***	***M***	***F***
**China**	***DFLE (SE)***	12.9 (0.3)	14.4 (0.3)	9.5 (0.3)	10.7 (0.3)	6.8 (0.3)	7.7 (0.3)	5.1 (0.2)	5.1 (0.3)	3.6 (0.2)	3.3 (0.3)
	%	91.2	89.0	88.1	85.9	83.7	81.3	81.9	73.5	79.0	64.0
**Cuba**	***DFLE (SE)***	15.8 (0.3)	16.8 (0.3)	12.4 (0.3)	13.1 (0.3)	9.3 (0.3)	9.8 (0.3)	6.7 (0.3)	6.8 (0.3)	4.8 (0.3)	4.3 (0.2)
	%	92.6	87.2	90.3	84.2	87.1	80.0	82.3	73.1	77.3	62.5
**Dominican**	***DFLE (SE)***	15.6 (0.4)	16.5 (0.4)	12.8 (0.4)	13.2 (0.3)	10.4 (0.3)	10.4 (0.3)	8.5 (0.3)	7.9 (0.3)	6.9 (0.3)	5.6 (0.3)
**Republic**	***%***	93.8	88.3	92.8	85.8	92.1	83.0	92.0	77.2	90.7	67.9
**India**	***DFLE (SE)***	12.0 (0.3)	12.3 (0.3)	9.4 (0.2)	9.5 (0.3)	7.1 (0.3)	7.1 (0.3)	5.3 (0.3)	5.0 (0.4)	3.8 (0.3)	4.0 (0.3)
	%	91.8	86.9	90.5	84.2	87.5	79.9	85.3	72.7	80.2	75.5
**Mexico**	***DFLE (SE)***	15.8 (0.4)	16.5 (0.4)	12.4 (0.3)	13.0 (0.4)	9.4 (0.3)	9.8 (0.3)	6.8 (0.3)	7.1 (0.3)	4.7 (0.3)	4.9 (0.3)
	%	92.1	88.4	90.0	86.0	87.7	82.8	83.7	79.4	77.2	72.2
**Peru**	***DFLE (SE)***	14.3 (0.4)	15.8 (0.3)	11.2 (0.3)	12.2 (0.3)	8.5 (0.3)	9.0 (0.3)	6.1 (0.3)	6.1 (0.3)	4.4 (0.2)	4.0 (0.3)
	%	91.7	88.9	89.9	86.0	87.5	81.6	83.6	74.1	78.3	65.6
**Puerto Rico**	***DFLE (SE)***	15.2 (0.5)	18.0 (0.4)	12.1 (0.3)	14.2 (0.3)	9.2 (0.3)	10.6 (0.3)	6.7 (0.3)	7.4 (0.3)	4.7 (0.3)	4.8 (0.3)
	%	91.3	88.1	89.7	85.8	87.0	81.7	82.6	75.1	75.3	65.6
**Venezuela**	***DFLE (SE)***	13.8 (0.4)	15.5 (0.5)	10.8 (0.4)	12.1 (0.5)	8.3 (0.4)	9.4 (0.5)	6.1 (0.5)	7.1 (0.5)	4.6 (0.4)	5.3 (0.4)
	%	91.8	83.9	90.0	79.8	87.1	75.0	81.5	69.1	77.4	63.7
